# Microbiota-derived indole-3-propionic acid regulates glucose homeostasis via remodeling of hepatic mitochondrial metabolism

**DOI:** 10.1080/19490976.2026.2760041

**Published:** 2026-11-06

**Authors:** Or Maalumi, Ziv Ben Moshe, Or Blank, Rachel Barkan-Michaeli, Avihu H. Yona, Kfir Sharabi

**Affiliations:** a The Institute of Biochemistry, Food Science, and Nutrition, The Robert H. Smith Faculty of Agriculture, The Hebrew University of Jerusalem, Rehovot, Israel

**Keywords:** Indole-3-propionic acid, hepatic glucose production, gluconeogenesis, gut microbiota, mitochondrial metabolism, glucagon

## Abstract

The gut microbiota produces metabolites that circulate to host tissues and are increasingly linked to metabolic health, yet the mechanisms by which individual microbial products regulate liver glucose metabolism remain poorly defined. Here, we identify the tryptophan-derived microbial metabolite indole-3-propionic acid (IPA) as a direct modulator of hepatic glucose production. In primary hepatocytes, a focused screen of indole metabolites revealed that several indole-containing compounds suppress glucagon-stimulated glucose output, with IPA selected for further characterization. IPA selectively reduced glucose production from mitochondrial-dependent gluconeogenic substrates while largely preserving glycerol-supported glucose production, suggesting that it does not simply shut down gluconeogenesis but instead alters how hepatocytes use metabolic fuels. Mechanistic analyzes showed that IPA redirects lactate-derived carbon away from glucose production and reshapes mitochondrial metabolism, including redox balance, ATP availability, and urea cycle-linked metabolic activity. These effects occurred without detectable disruption of proximal insulin or glucagon signaling, supporting a model in which IPA acts primarily through intracellular metabolic remodeling. In mice, endogenous IPA levels varied with nutritional state, and short-term IPA administration improved fasting glycemia and glucose handling in Western diet-fed animals. Finally, microbiome-depleted mice colonized with IPA-producing *Clostridium sporogenes* displayed increased circulating IPA and improved glucose tolerance compared with mice colonized with an IPA-deficient mutant *C. Sporogenes* strain. Together, these findings identify IPA as a microbial metabolite that directly connects gut tryptophan metabolism to hepatic mitochondrial function and systemic glucose regulation, highlighting a mechanistic gut-liver pathway with potential therapeutic relevance to metabolic disease.

## Graphical abstract

**Figure:**
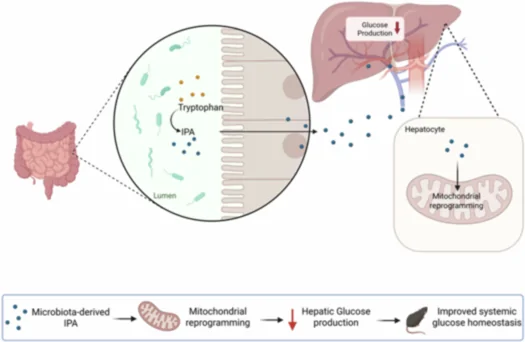

## Introduction

Maintenance of fasting blood glucose levels relies predominantly on hepatic glucose production (HGP), a process tightly regulated by the opposing actions of insulin and glucagon.^1-3^ In insulin-resistant states, including metabolic dysfunction-associated steatotic liver disease (MASLD) and type 2 diabetes, suppression of HGP is impaired, leading to excessive hepatic glucose output and fasting hyperglycemia.^4^ ^,^ ^5^ While transcriptional induction of gluconeogenic enzymes contributes to this dysregulation, accumulating evidence indicates that acute, post-translational, and metabolic control mechanisms play a major role in determining gluconeogenic flux.^6^ ^,^ ^7^ Glucagon is a dominant driver of HGP during fasting and in insulin-resistant conditions, acting through cAMP-dependent signaling pathways to stimulate gluconeogenesis and hepatic energy mobilization. Importantly, glucagon-stimulated glucose production depends not only on canonical signaling cascades but also on the metabolic capacity of hepatocytes to support sustained carbon flux through gluconeogenic pathways.^8-10^ How this metabolic capacity is modulated in physiological and pathophysiological contexts remains incompletely understood.

The gut microbiota has emerged as a critical regulator of host metabolic homeostasis through the production of bioactive metabolites that enter the systemic circulation.^11^ ^,^ ^12^ These metabolites, derived from dietary components and microbial metabolic activity, can influence glucose and lipid metabolism, inflammation, and energy balance across multiple organs.^13^ Because the liver is directly exposed to gut-derived metabolites via the portal vein, hepatocytes are uniquely positioned to integrate microbial signals into metabolic responses.^14^ Despite growing recognition of microbiota-host crosstalk, the mechanisms by which individual microbial metabolites influence hepatic metabolic pathways remain poorly defined. In particular, whether these metabolites exert their effects primarily through receptor-mediated signaling, transcriptional reprogramming, or direct modulation of intracellular metabolic processes is often unclear. Dissecting these mechanisms is essential for understanding how gut microbial activity contributes to metabolic health and disease.

Indole metabolites constitute a chemically related group of microbiota-derived compounds produced from dietary tryptophan and present in the systemic circulation.^15^ ^,^ ^16^ Several indoles reach the liver via the portal vein, positioning hepatocytes as direct targets of microbial metabolic activity.^15^ Within this class, indole-3-propionic acid (IPA) has emerged as a prominent metabolite due to its abundance, microbial origin,^17^ ^,^ ^18^ and consistent association with favorable metabolic phenotypes. A recent study reported lower circulating IPA concentrations in individuals with type 2 diabetes than in healthy controls, together with inverse associations between IPA and HbA1c, fasting glucose, and glycemic variability.^19^ A seven-day broad-spectrum antibiotic intervention reduced circulating IPA to near-undetectable levels in both groups, providing direct human evidence for its predominantly microbial origin.^19^ In experimental models, IPA improves glucose metabolism in rats,^20^ protects intestinal barrier function through PXR- and TLR4-linked mechanisms,^21^ attenuates diet-induced steatohepatitis in rats by reducing gut dysbiosis and endotoxin leakage,^22^ and protects against heart failure with preserved ejection fraction, in part through effects on NAD metabolism.^23^ More recent studies further support the idea that IPA can act through metabolic remodeling: IPA promotes mitochondrial respiration in CD4+ T cells to confer protection against intestinal inflammation,^24^ and microbiota-derived IPA promotes mucosal healing in colitis through intestinal HMGCS2-dependent ketogenesis.^25^ Together, these studies position IPA as a bioactive microbial metabolite capable of influencing host metabolism, mitochondrial function, inflammatory tone, and tissue homeostasis across multiple physiological contexts.

Despite this growing body of evidence, the direct hepatic actions of IPA remain insufficiently defined. Prior studies in metabolic disease models have largely emphasized systemic, intestinal, inflammatory, or microbiome-mediated mechanisms, whereas it remains unclear whether IPA can act directly on hepatocytes to regulate glucose production. This distinction is particularly important because hepatic glucose output is acutely controlled by hormone-driven metabolic flux, not only by longer-term transcriptional or inflammatory remodeling. Although IPA has been implicated in signaling through host receptors such as the aryl hydrocarbon receptor and pregnane X receptor,^21^ ^,^ ^26^ ^,^ ^27^ and in the modulation of mitochondrial or NAD-linked metabolism in other cell types,^23^ ^,^ ^24^ whether these mechanisms converge on hepatocyte gluconeogenic control during glucagon stimulation has not been established. Specifically, it remains unknown whether IPA influences hepatic glucose output through altered hormone signaling, changes in substrate handling, or direct remodeling of intracellular metabolic state.

Hepatic glucose production is ultimately constrained by mitochondrial metabolic capacity, which integrates hormonal cues with intracellular redox balance, energy state, and carbon flux through central metabolic pathways. During glucagon stimulation, efficient mitochondrial function is required to sustain gluconeogenic flux even in the presence of intact upstream signaling. This study was motivated by previous work identifying SR18292 as a small-molecule suppressor of glucagon-induced hepatic glucose production,^28^ followed by structure-activity analyzes demonstrating that its indole-containing chemical scaffold is required for this biological activity.^29^ These findings raised the possibility that structurally related indole-containing metabolites, including microbiota-derived indoles, may also influence hepatic glucose production. We therefore performed a targeted screen of selected indole metabolites and identified IPA as a biologically relevant suppressor of glucagon-stimulated glucose production. We then used mechanistic analyzes in primary hepatocytes, including metabolic profiling, isotope tracing, and measurements of cellular redox and energy status, to define how IPA alters hepatocyte metabolic function under glucagon-stimulated conditions. These in vitro studies were complemented by acute in vivo IPA treatment in mice to assess the physiological relevance of our findings for whole-body glucose homeostasis. Together, this integrated approach was designed to determine whether IPA functions as a microbiota-derived effector linking gut microbial metabolism to hepatic glucose regulation.

## Results

### Indole derivatives suppress hepatic glucose production in a substrate-selective manner

Guided by prior structure-activity relationship studies demonstrating that the indole moiety of SR-18292 is required for its ability to suppress hepatic glucose production,^28^ ^,^ ^29^ we asked whether structurally related indole-containing metabolites can directly influence hepatocyte glucose output. To test this, we performed a focused screen of selected indole derivatives (Figure 1A) and measured glucagon-stimulated glucose production in primary hepatocytes using pyruvate/lactate or glycerol as gluconeogenic substrates. Several indole derivatives significantly reduced glucose production from pyruvate/lactate, whereas glucose production from glycerol was less consistently affected across the compound panel (Figure 1B). These findings indicate that indole derivatives do not act as broad inhibitors of gluconeogenic capacity, but instead suppress glucose production in a substrate-selective manner.

**Figure 1.**
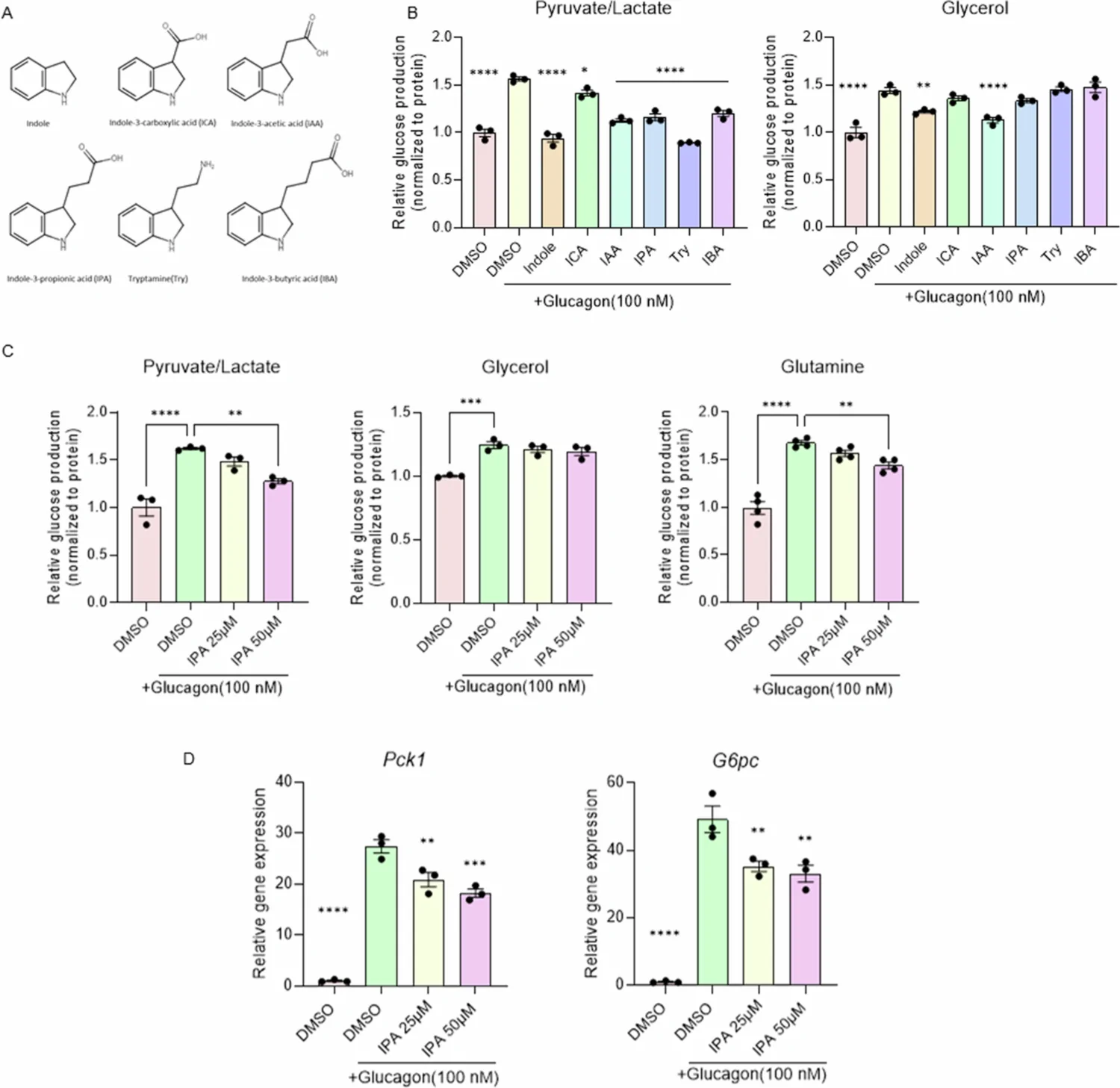
Indole-derived metabolites suppress hepatic glucose production. (A) Chemical structures of the indole derivatives tested. (B) Glucose production by primary mouse hepatocytes treated with the indicated indole derivatives at 50 μM. Cells were pretreated for 3 h, followed by a 6-h glucose-production assay with glucagon (100 nM), using either pyruvate/lactate (2/20 mM) or glycerol (10 mM) as substrates. Statistical comparisons were made against the vehicle-plus-glucagon group within each substrate condition. (C) Glucose production following treatment with IPA, using pyruvate/lactate (2/20 mM), glycerol (10 mM), or glutamine (4 mM) as substrates. (D) Relative *Pck1* and *G6pc* mRNA expression measured by RT-qPCR following 3 h of IPA pretreatment and an additional 3 h of glucagon stimulation (100 nM). Data are presented as mean ± SEM. We assessed statistical significance by ANOVA followed by Dunnett’s multiple-comparisons test. Significance symbols denote multiplicity-adjusted *P* values. *, *P* < 0.05; **, *P* < 0.01; ***, *P* < 0.001; ****, *P* < 0.0001.

Among the compounds tested, we prioritized indole-3-propionic acid (IPA) because of its microbial origin and repeated associations with favorable glucose metabolism, including a lower risk of developing type 2 diabetes in prospective human studies.^19^ ^,^ ^30-32^ Although other derivatives produced greater inhibition under the screening conditions, IPA largely preserved glycerol-supported glucose production, providing an opportunity to investigate its substrate-selective metabolic actions. Dose-response experiments confirmed that IPA suppresses glucagon-stimulated glucose production from pyruvate/lactate, while glucose production from glycerol was largely preserved over the same concentration range (Figure 1C). In the expanded dose-response analysis (1–100 μM), statistically significant suppression was detected at 25 μM, with greater inhibition at 50–100 μM (Fig. S1B). Notably, IPA also reduced glucose production from glutamine, particularly at the higher concentration tested (Figure 1C). Thus, IPA preferentially suppresses glucose production from substrates that require mitochondrial metabolism or anaplerotic processing before entering the gluconeogenic pathway, while sparing glycerol-supported glucose production. Consistent with the reduction in glucose output, IPA also dose-dependently attenuated glucagon-induced expression of the canonical gluconeogenic genes *Pck1* and *G6pc* (Figure 1D). These data indicate that IPA can suppress both functional glucose production and selected components of the glucagon-induced gluconeogenic transcriptional program. However, because glycerol-supported glucose production was largely preserved despite reduced *Pck1* and *G6pc* expression, the functional effect of IPA cannot be explained solely by broad transcriptional repression of gluconeogenesis. We next examined whether this transcriptional effect extended more broadly across the indole derivative panel. Glucagon robustly induced *G6pc* and *Pck1* expression, and several indole derivatives attenuated this response, with the most consistent suppression observed for *Pck1* (Figure S1A). In contrast, effects on *G6pc* were more variable, and *Ppargc1a* expression was not uniformly reduced across the compound panel. These data indicate that indole derivatives can partially suppress selected components of the glucagon-responsive gluconeogenic gene program, but do not cause a broad shutdown of glucagon-induced transcription.

We assessed cell viability using an MTT assay. IPA did not reduce the MTT signal at concentrations up to 500 μM, providing no evidence of reduced viability under the conditions tested (Figure S1C). However, because MTT reduction reflects cellular metabolic activity, this assay does not definitively exclude cytotoxicity. We next asked whether IPA's effect could be explained by altered proximal hormone signaling. IPA did not reduce insulin-stimulated AKT phosphorylation, indicating that canonical insulin signaling remained intact (Figure S1D). Similarly, IPA did not blunt glucagon-induced CREB phosphorylation or AMPK phosphorylation, suggesting that suppression of glucose production is not due to inhibition of proximal glucagon-CREB or AMPK signaling (Figure S1E). Together, these findings support the idea that IPA acts downstream of, or parallel to, canonical hormone receptor signaling.

IPA has also been reported to signal through host xenobiotic receptors, including the aryl hydrocarbon receptor (AhR) and the pregnane X receptor (PXR/*Nr1i2*),^21^ ^,^ ^26^ ^,^ ^27^ which mediate some of its biological effects in other tissues and experimental systems.^21^ ^,^ ^26^ We therefore tested whether these pathways are required for IPA-mediated suppression of glucose production. The IPA-associated reductions in glucagon-induced *Pck1* and *G6pc* expression were attenuated and did not reach statistical significance in the presence of the AhR inhibitor CH223191 (Figure S2B), whereas suppression of glucose production persisted (Figure S2A). In contrast, IPA continued to suppress both transcripts following knockdown of *Nr1i2*, the gene encoding PXR (Figure S2D). These findings distinguish the transcriptional and functional responses and suggest that reduced *Pck1*/*G6pc* expression alone does not explain IPA-mediated suppression of glucose output. Thus, the functional effect persisted during pharmacological AhR inhibition and PXR knockdown under the conditions tested.

Together, these data identify IPA as a microbiota-derived indole metabolite that suppresses glucagon-stimulated hepatic glucose production in a substrate-selective manner. The preferential effect on pyruvate/lactate-driven glucose output, together with preserved glycerol-supported glucose production and largely intact proximal hormone signaling, suggested that IPA does not simply inhibit gluconeogenesis globally. Instead, these findings pointed to a more specific effect on the handling of lactate-derived carbon during glucagon-stimulated glucose production.

### IPA remodels lactate-derived carbon flux at the TCA-urea cycle interface

Having established that IPA suppresses glucagon-stimulated glucose production from pyruvate/lactate, we next asked whether this phenotype reflects altered handling of lactate-derived carbon. To address this, we performed metabolic tracing using uniformly labeled [U-^13^C]-lactate in primary hepatocytes under basal and glucagon-stimulated conditions. Consistent with the reduction in glucose output observed functionally, IPA significantly reduced the fraction of ^13^C₃-labeled glucose-6-phosphate (G6P), particularly under glucagon-stimulated conditions (Figure 2A, B). This indicates that IPA limits the incorporation of lactate-derived carbon into the gluconeogenic pathway.

**Figure 2.**
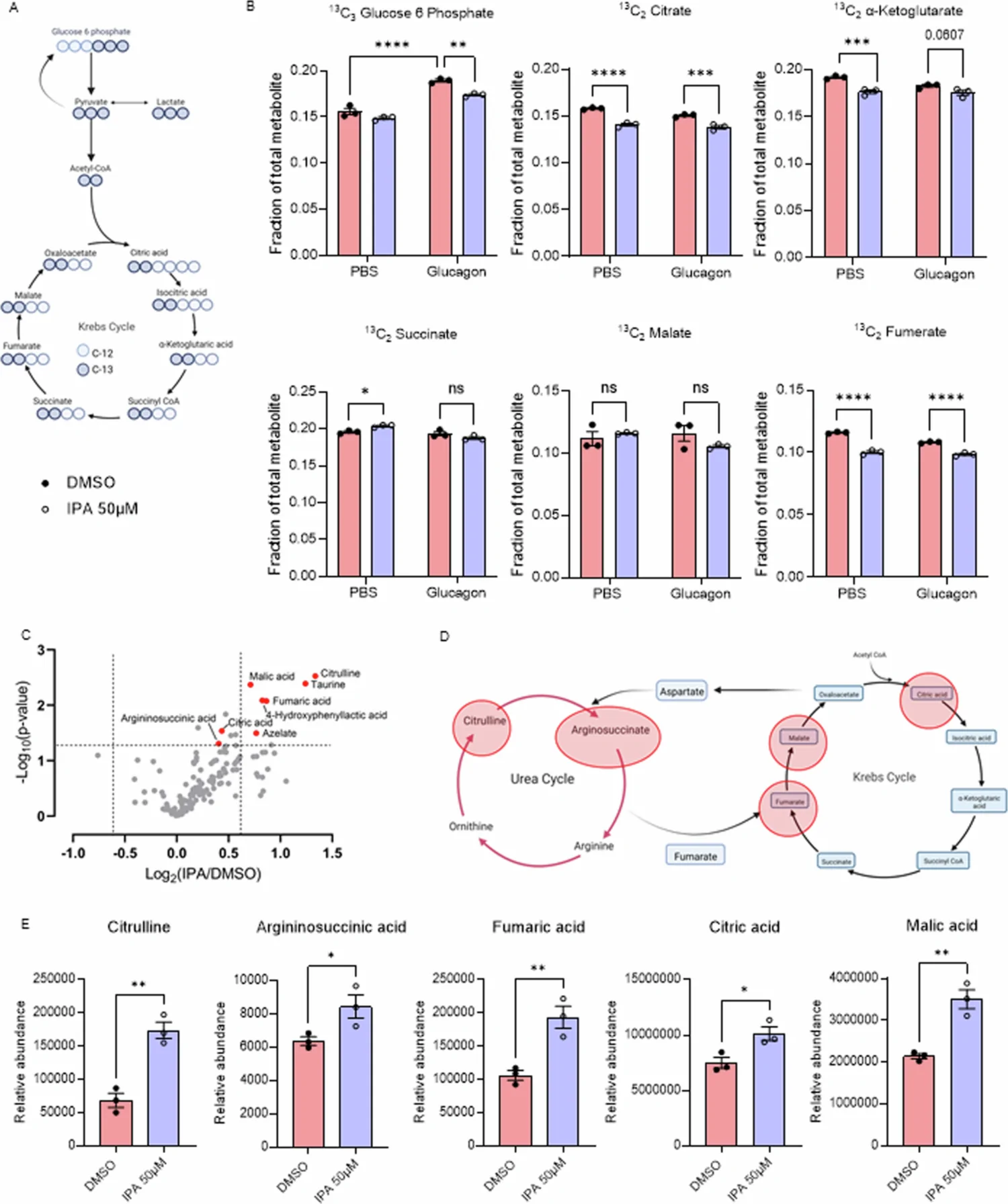
IPA alters lactate-derived carbon labeling and TCA-urea cycle-associated metabolite pools. (A) Schematic illustrating incorporation of [U-^13^C]lactate-derived carbon into glucose-6-phosphate (G6P) and TCA cycle intermediates. (B) Fractional enrichment of M + 3 G6P and M + 2 TCA cycle intermediates in primary mouse hepatocytes. Cells were pretreated with IPA (50 μM) or DMSO for 3 h, followed by a 3-h incubation with [U-^13^C]lactate in the absence or presence of glucagon (100 nM), with the corresponding IPA or vehicle treatment maintained. (C–E) Polar metabolite analysis of primary hepatocytes treated with IPA (50 μM) or DMSO for 3 h under basal conditions without glucagon. (C) Volcano plot showing log_2_ fold changes in metabolite abundance and −log^10^ *P* values. (D) Schematic linking the TCA and urea cycles, with metabolites increased by IPA highlighted in red. (E) Relative abundances of selected metabolites. Data are presented as mean ± SEM; *n* = 3 per condition. Statistical comparisons were performed using two-way ANOVA with IPA treatment and glucagon stimulation as factors, followed by Sidak’s multiple-comparisons test (B), or unpaired t-tests comparing IPA with DMSO (C, E). *P* values are adjusted for multiple comparisons in B and nominal in C and E. *, *P* < 0.05; **, *P* < 0.01; ***, *P* < 0.001; ****, *P* < 0.0001; ns, not significant.

We then examined whether IPA also alters the propagation of lactate-derived carbon through mitochondrial metabolic intermediates. Across several TCA cycle metabolites, including citrate, *α*-ketoglutarate, malate, and fumarate, IPA reduced fractional ^13^C₂ enrichment from lactate in both basal and glucagon-stimulated states (Figure 2B), indicating a lower relative contribution of lactate-derived carbon to these metabolite pools. Succinate did not change significantly under these conditions. This coordinated reduction across multiple intermediates argues against inhibition at a single enzymatic step and instead suggests that IPA broadly alters the entry or retention of lactate-derived carbon within mitochondrial carbon metabolism. Notably, IPA reduced labeling of malate and fumarate, intermediates positioned at the interface between the TCA cycle, gluconeogenesis, and nitrogen metabolism, suggesting that IPA affects metabolic nodes directly relevant to lactate-supported glucose production.

To determine whether IPA also induces broader metabolic remodeling independent of acute hormonal stimulation, we performed polar metabolite analysis in primary hepatocytes treated with IPA for 3 h under basal conditions. Volcano plot analysis revealed a focused set of metabolites increased by IPA rather than a global shift across the metabolome (Figure 2C). Pathway mapping showed that the most responsive metabolites clustered at the intersection of mitochondrial carbon metabolism and nitrogen handling (Figure 2D). IPA increased the abundance of urea cycle-associated intermediates, including citrulline and argininosuccinic acid, together with connected TCA cycle intermediates, including fumaric acid, malic acid, and citric acid (Figure 2E). Citrulline showed the largest increase, while fumarate and malate were also prominently elevated. Importantly, these steady-state metabolite changes were observed in the absence of glucagon stimulation, indicating that IPA induces an early reorganization of hepatocyte metabolic state rather than simply blocking glucagon signaling. When considered together with the isotope-tracing data, the accumulation of selected TCA-urea cycle intermediates despite reduced fractional labeling from ^13^C-lactate indicates that IPA alters the relative contribution of lactate-derived carbon to these metabolite pools. Because fractional enrichment is influenced by both labeled input and total pool size, these data do not distinguish whether the expanded pools reflect increased contribution from unlabeled substrates, altered metabolite turnover, or other changes in pathway dynamics. Thus, IPA reduces the fractional contribution of lactate-derived carbon to gluconeogenic and mitochondrial intermediates while selectively remodeling metabolite pools at the TCA–urea cycle interface.

Together, these findings provide a metabolic explanation for the substrate-selective suppression of glucose production observed in Figure 1. IPA does not appear to broadly inhibit gluconeogenesis, but rather alters the metabolic routing of lactate-derived carbon through pathways that support glucagon-stimulated glucose production.

### IPA reshapes mitochondrial redox state and urea cycle-linked metabolism during glucagon stimulation

Consistent with the reduced incorporation of lactate-derived carbon into gluconeogenic and mitochondrial intermediates observed in the ^13^C-lactate tracing experiments above, we next asked whether IPA alters hepatocyte redox state and energy balance during glucagon stimulation. IPA treatment increased the cellular NAD⁺/NADH redox balance relative to vehicle control (Figure 3A,B). Glucagon stimulation similarly elevated the NAD⁺/NADH redox balance, consistent with activation of oxidative metabolism during gluconeogenic stimulation. However, the combination of IPA and glucagon did not produce a further increase and instead shifted the ratio toward baseline levels, indicating that IPA modifies the redox response to glucagon rather than simply amplifying mitochondrial oxidation.

**Figure 3.**
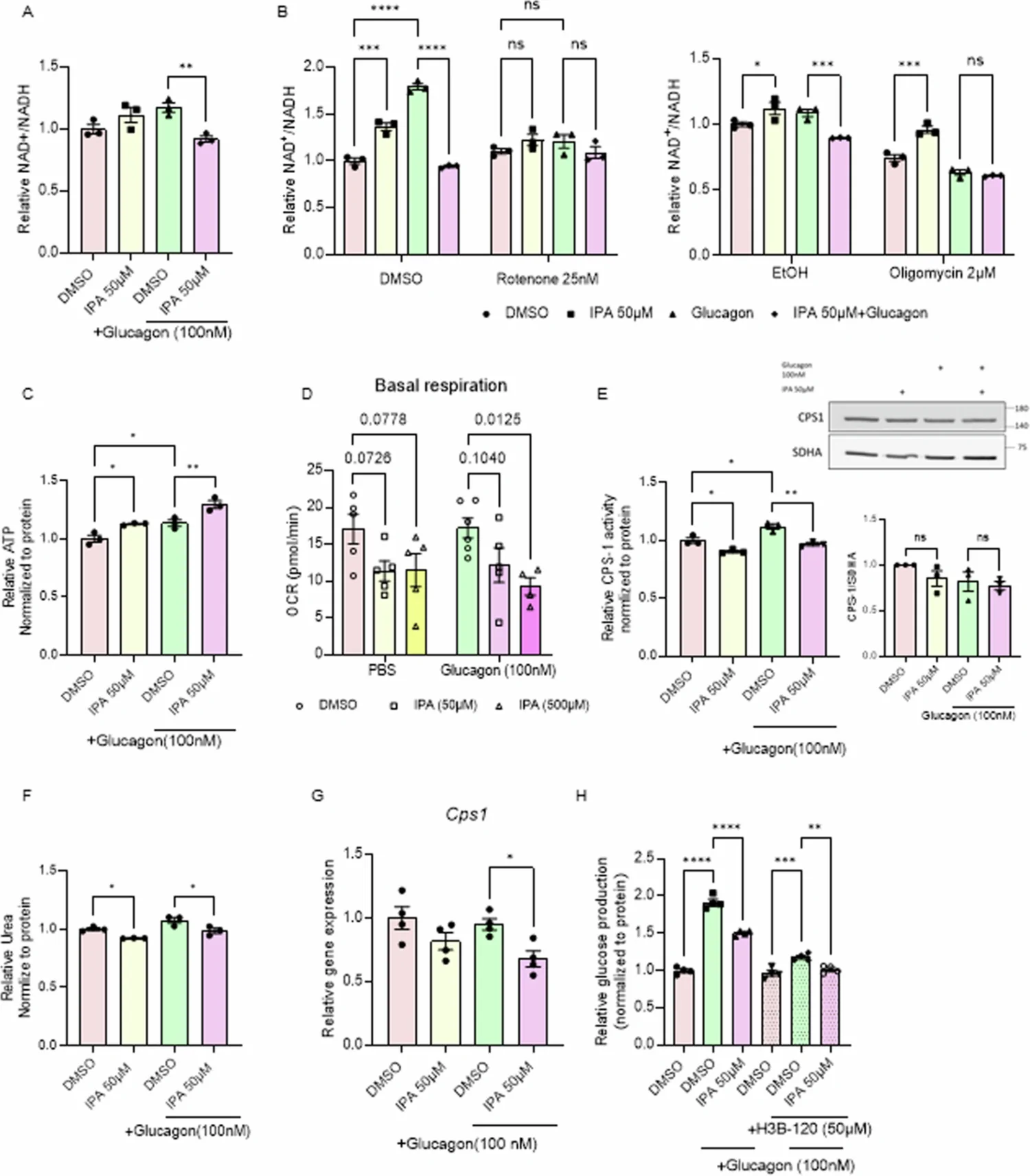
IPA regulates hepatic redox and ureagenesis. Primary mouse hepatocytes were treated with IPA (50 μM) or DMSO, with or without glucagon (100 nM), unless otherwise indicated. (A) Cellular NAD⁺/NADH ratio following 3 h of IPA pretreatment and an additional 3 h with or without glucagon. (B) NAD⁺/NADH ratio under the same conditions, with rotenone (25 nM; Complex I inhibitor), oligomycin (2 μM; ATP synthase inhibitor), or the corresponding vehicle added during the final hour. (C) Cellular ATP abundance normalized to total protein. (D) Basal oxygen consumption rate measured by Seahorse respirometry following 3 h of pretreatment with IPA (50 or 500 μM) and an additional 2 h with or without glucagon. OCR was normalized to cell number. (E) CPS1 activity measured in mitochondrial preparations isolated following 3 h of IPA pretreatment and an additional 3 h with or without glucagon. Activity was assayed under defined conditions containing ATP and *N*-acetylglutamate and normalized to mitochondrial protein. Representative immunoblots and quantification show CPS1 abundance relative to SDHA. (F) Urea released into the medium during a 6-h incubation with or without glucagon following 3 h of IPA pretreatment, normalized to total cellular protein. (G) Relative *Cps1* mRNA expression measured by RT-qPCR following 3 h of IPA pretreatment and an additional 3 h with or without glucagon. (H) Glucose production from pyruvate/lactate (2/20 mM) during a 6-h assay in the presence or absence of IPA and the CPS1 inhibitor H3B-120 (50 μM), normalized to total cellular protein. Statistical analyzes were performed using one-way ANOVA (A, C, E–H) or two-way ANOVA (B, D), followed by Sidak’s post hoc analysis for multiple comparisons. Numerical *P* values are shown in D. *, *P* < 0.05; **, *P* < 0.01; ***, *P* < 0.001; ****, *P* < 0.0001; ns, not significant.

To determine whether these redox effects depend on mitochondrial respiration, we repeated these measurements in the presence of respiratory inhibitors. Inhibition of Complex I with rotenone abolished the IPA-induced increase in NAD⁺/NADH redox balance and also prevented the glucagon-induced increase observed under control conditions (Figure 3B). In the presence of rotenone, neither glucagon nor glucagon+IPA altered the NAD⁺/NADH ratio relative to basal conditions (Figure 3B). Thus, both the IPA-associated and the glucagon-associated redox responses require intact rotenone-sensitive mitochondrial NADH oxidation. Because rotenone blocks the entry point for NADH into the respiratory chain, this experiment shows that these redox shifts depend on ongoing Complex I-linked electron transport, but it does not indicate where within mitochondrial metabolism IPA acts, nor does it identify Complex I as an IPA target. Notably, IPA did not raise NAD⁺/NADH under rotenone, arguing against a mechanism acting on cytosolic NAD⁺ regeneration or on NADH-consuming reactions independent of the respiratory chain. In contrast, inhibition of ATP synthase with oligomycin did not prevent the IPA-induced increase in NAD⁺/NADH redox balance under basal conditions, but it abolished the glucagon-associated increase in NAD⁺/NADH and eliminated the effect of IPA under glucagon stimulation (Figure 3B). Thus, the basal redox effect of IPA can still occur when ATP synthase is inhibited, whereas the glucagon-dependent redox response is sensitive to ATP synthase inhibition. In parallel, IPA increased cellular ATP levels under both basal and glucagon-stimulated conditions (Figure 3C), indicating that IPA does not broadly impair mitochondrial energy production. Because ATP increased in both conditions whereas the NAD⁺/NADH response differed between basal and glucagon-stimulated states, ATP levels do not simply mirror the redox ratio. Rather, these data suggest that IPA alters mitochondrial energy handling together with Complex I-dependent redox balance, with glucagon modifying the way this redox-energy state is expressed.

Because rotenone abolished the IPA-associated increase in NAD⁺/NADH, we next tested whether IPA alters mitochondrial respiration directly using Seahorse respirometry. IPA changed hepatocyte OCR in a concentration-dependent manner. At 50 μM, IPA produced modest changes in basal OCR that did not reach statistical significance, whereas higher-dose IPA significantly reduced basal respiration, particularly under glucagon-stimulated conditions (Figure 3D). These findings indicate concentration-dependent effects on hepatocyte respiration, but do not establish respiratory inhibition as the mechanism underlying suppression of glucose production at 50 μM or identify Complex I as a direct molecular target. Importantly, the increased ATP abundance (Figure 3C) does not necessarily indicate increased mitochondrial ATP production, as cellular ATP levels reflect the balance between production and utilization. The absence of a significant increase in OCR at 50 μM IPA suggests that the higher ATP levels are unlikely to result simply from increased oxidative phosphorylation. One possibility is that suppression of ATP-demanding metabolic processes, including gluconeogenesis, contributes to ATP accumulation, particularly during glucagon stimulation. Thus, the ATP and respirometry data together indicate that IPA alters cellular energy balance rather than uniformly stimulating mitochondrial respiration.

Because the metabolomics data pointed to remodeling at the interface between mitochondrial carbon metabolism and the urea cycle, we next examined whether IPA also affects CPS1-dependent urea cycle function. Glucagon modestly increased CPS1 activity, whereas IPA attenuated CPS1 activity most clearly under glucagon-stimulated conditions (Figure 3E). IPA also reduced urea secretion in glucagon-stimulated hepatocytes (Figure 3F), supporting a functional reduction in urea cycle output. Although *Cps1* mRNA expression was reduced in the IPA-treated cells (Figure 3G), total CPS1 protein levels were not detectably decreased (Figure 3E), indicating that the reduction in CPS1 activity is unlikely to be driven simply by reduced CPS1 abundance. Notably, we measured CPS1 activity in mitochondria isolated from treated hepatocytes and assayed it in a defined buffer containing saturating *N*-acetylglutamate and ATP, along with bicarbonate and ammonium. Under these conditions, the intracellular metabolic environment has been removed, and none of the substrates or the allosteric activator is limiting. The reduced activity we observe therefore reflects a property of the enzyme that persists through mitochondrial isolation, rather than acute limitation of substrate or cofactor availability in the intact cell.

Finally, pharmacological inhibition of CPS1 with H3B-120 reduced glucagon-stimulated glucose production, indicating that CPS1-dependent mitochondrial nitrogen metabolism contributes to the metabolic capacity required for maximal glucose output (Figure 3H). However, IPA further reduced glucose production in the presence of H3B-120, although the magnitude of the IPA effect was smaller than in its absence (Figure 3H). Thus, while CPS1 inhibition partially phenocopies the effect of IPA, the residual IPA sensitivity under CPS1-inhibited conditions suggests that IPA does not act solely through CPS1. Rather, CPS1-dependent urea cycle activity appears to represent one component of a broader IPA-sensitive mitochondrial metabolic state that supports lactate-dependent glucose production.

Together, these data indicate that IPA remodels the glucagon-stimulated mitochondrial state by altering Complex I-dependent redox balance, ATP availability, and CPS1-linked nitrogen metabolism, while limiting lactate-dependent hepatic glucose production through mechanisms that are only partly explained by reduced CPS1 activity.

### Endogenous IPA levels vary with nutritional state, and short-term IPA administration improves glucose handling in WD-fed mice

Given that IPA is a microbiota-derived metabolite generated from dietary tryptophan, we first examined whether systemic IPA availability varies across physiological nutritional states. In chow-fed mice with an intact microbiome (i.e., without antibiotic depletion), targeted LC-MS/MS analysis revealed that circulating and hepatic IPA levels fluctuate with feeding status. Both serum and liver IPA concentrations differed between ad libitum, overnight fasted (16 h), and refed (2 h) conditions (Figure 4A), consistent with the idea that endogenous IPA availability dynamically reflects nutrient intake and microbial metabolism of dietary tryptophan. To determine whether circulating IPA changes following a metabolic challenge, we repeated this experiment in a new cohort following Western diet (WD) feeding. Again, when mice were fed a chow diet, refeeding markedly increased serum IPA (Figure 4B); however, when the same mice were fed WD for ~6 weeks, both basal and refed serum IPA were reduced substantially, and no increase in serum IPA was observed in response to refeeding.

**Figure 4.**
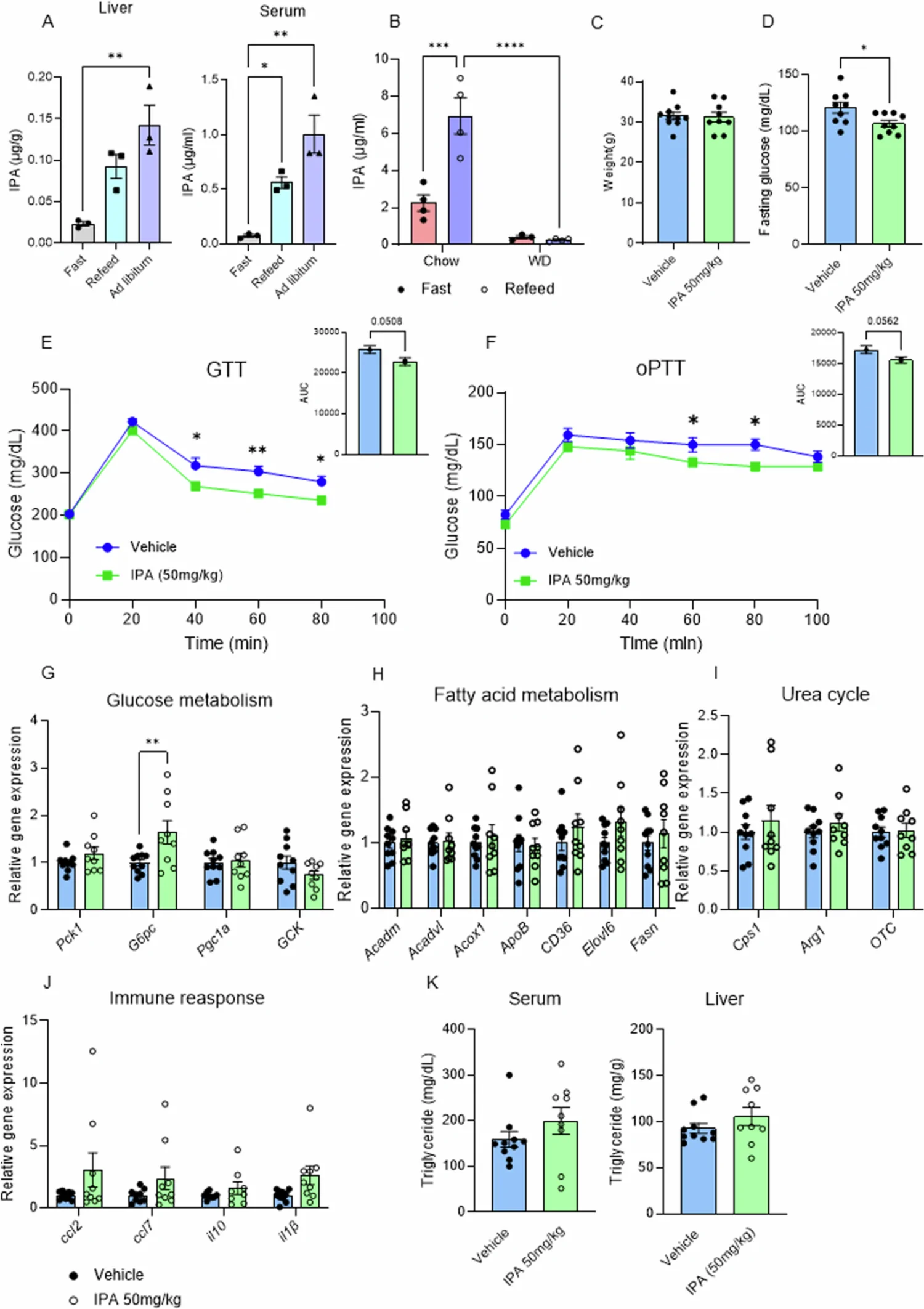
Acute IPA treatment improves metabolic homeostasis in vivo. (A) Serum and hepatic IPA concentrations in chow-fed mice under ad libitum feeding, following a 16 h fast, or following 2 h of refeeding after fasting (*n* = 3 per condition). (B) Serum IPA concentrations following overnight fasting or 2 h of refeeding, measured in the same mice during chow feeding and subsequently after 4 weeks of Western diet (WD) feeding (*n* = 4). (C–K) Mice maintained on WD for approximately 8 weeks received IPA (50 mg/kg, i.p.) or vehicle once daily for 5 days. (C) Body weight (*n* = 10/9). (D) Fasting blood glucose (*n* = 10/9). (E) Intraperitoneal glucose tolerance test (GTT; 2 g/kg glucose) following overnight fasting, with area under the curve (AUC) shown in the inset (*n* = 7/7). (F) Oral pyruvate tolerance test (oPTT; 2.5 g/kg pyruvate) following overnight fasting, with AUC shown in the inset (*n* = 10/10). Fasting glycemia, GTT, and oPTT were assessed in separate mouse cohorts. (G–J) Relative hepatic mRNA expression of genes involved in glucose metabolism (G), fatty acid metabolism (H), the urea cycle (I), and immune responses (J), measured by RT-qPCR. (K) Serum and hepatic triglyceride concentrations. For G–K, samples were collected following overnight fasting, 2 h after the final IPA or vehicle injection. Data are presented as mean ± SEM. Individual points represent individual mice. Statistical analyzes were performed using one-way ANOVA followed by Tukey’s multiple-comparisons test (A), two-way ANOVA followed by Sidak’s multiple-comparisons test (B, G–J), unpaired t-tests with Welch’s correction (C, D, K), or multiple unpaired t-tests comparing treatment groups at individual time points (E–F). Numerical *P* values are shown for the AUC comparisons in E and F. *, *P* < 0.05; **, *P* < 0.01; ***, *P* < 0.001; ****, *P* < 0.0001.

Having established that IPA alters hepatocyte metabolic state and suppresses glucose production in vitro, we next asked whether increasing systemic IPA exposure influences glucose metabolism in vivo. Because WD feeding markedly reduced endogenous circulating IPA, we next measured serum IPA following i.p. IPA administration in WD-fed mice. Approximately 2 h after IPA administration to overnight-fasted WD-fed mice, serum IPA reached approximately 300 μg/mL (~1.6 mM), substantially exceeding endogenous circulating concentrations (Fig. S3A). Thus, the 50 mg/kg intervention represents acute pharmacological IPA exposure rather than restoration of circulating IPA to endogenous levels. Mice maintained on a WD for ~8 weeks, also with an intact microbiome, were then treated with IPA (50 mg/kg, i.p.) once daily for 5 days. Body weight remained unchanged during the treatment period (Figure 4C), indicating no overt effects on energy balance. In contrast, fasting blood glucose was reduced in IPA-treated mice compared with vehicle-treated controls (Figure 4D). During glucose tolerance testing, IPA-treated mice displayed reduced glycemic excursion, resulting in a lower GTT AUC (Figure 4E). During pyruvate tolerance testing, IPA-treated mice also showed a reduced glucose excursion, reflected by a lower PTT AUC (Figure 4F). These findings indicate that short-term IPA administration improves glucose handling in WD-fed mice and is associated with reduced pyruvate-driven glycemic excursion in vivo.

We next examined whether these physiological effects were accompanied by coordinated transcriptional remodeling of hepatic metabolic pathways. IPA treatment did not produce broad suppression of the hepatic gluconeogenic program (Figure 4G). Instead, expression of several glucose metabolism genes was largely unchanged, while *G6pc* expression was increased (Figure 4G). Genes involved in fatty acid metabolism were also mostly unchanged (Figure 4H), indicating that short-term IPA exposure does not broadly reprogram hepatic lipid metabolic gene expression under these conditions. Similarly, urea-cycle and inflammatory genes (Figure 4I,J) did not show a coordinated transcriptional pattern consistent with pathway-wide induction or suppression. Serum and hepatic triglyceride levels were not markedly altered (Figure 4K), further suggesting that the short-term metabolic effects of IPA were not secondary to major changes in hepatic lipid accumulation or systemic triglyceride availability. Notably, this in vivo transcriptional profile differs from the suppression of glucagon-responsive gluconeogenic gene expression observed in primary hepatocytes treated with IPA (Figure 1D). Thus, repression of the gluconeogenic transcriptional program is unlikely to represent the dominant mechanism underlying the in vivo phenotype. Instead, the reduction in fasting glycemia, improved glucose tolerance, and reduced PTT AUC are more consistent with changes in hepatic metabolic state or flux regulation rather than broad transcriptional reprogramming. Together with the in vitro data, these findings support a model in which IPA influences systemic glucose homeostasis, at least in part, by modulating hepatic metabolic function rather than by globally suppressing metabolic or inflammatory gene-expression programs.

### *fldC*-dependent *C. sporogenes* metabolism is associated with improved glucose tolerance following microbiome depletion

To examine the metabolic consequences of *fldC*-dependent bacterial metabolism, we compared wild-type and *fldC*-deficient *C. sporogenes* using a microbiome-depletion and recolonization approach.^33^ Antibiotic-treated chow-fed mice were colonized with either wild-type *C. sporogenes* or an isogenic *fldC*-deficient strain that is impaired in IPA production, and glucose homeostasis was assessed following colonization (Figure 5A). The microbiota-depletion model was used intentionally as a reductionist system to examine the metabolic consequences of introducing *C. sporogenes* with or without an intact *fldC*-dependent aromatic amino-acid pathway, rather than as a model of an intact microbial ecosystem. We administered both strains repeatedly throughout the experiment, rather than relying on a single inoculation followed by stable colonization. PCR analysis of stool samples confirmed the presence of the administered *C. sporogenes* strains (Fig. S4A), although this qualitative assessment does not establish equivalent colonization efficiency between the WT and *fldC*-deficient groups. Consistent with the expected metabolic difference between the two strains, mice colonized with wild-type *C. sporogenes* exhibited higher circulating IPA levels than mice colonized with the *fldC*-deficient strain (Figure 5B), while body weight was comparable between groups (Figure 5C). Functionally, wild-type *C. sporogenes* colonization produced a modest but detectable improvement in glucose tolerance, reflected by lower glycemic excursion during GTT and a reduced GTT AUC compared with mice receiving the *fldC*-deficient strain (Figure 5D). In contrast, pyruvate tolerance was largely unchanged between groups, with no clear reduction in PTT (Figure 5E). Thus, under chow-fed conditions, treatment with wild-type *C. sporogenes* was associated with higher circulating IPA and modestly improved glucose tolerance compared with the *fldC*-deficient strain, without a clear improvement in overall pyruvate tolerance. To determine whether the metabolic effects associated with *C. sporogenes fldC*-dependent metabolism are also observed under metabolic stress, we repeated the recolonization paradigm in WD-fed mice (Fig. S4B). Body weight was comparable between mice receiving WT or *fldC*-deficient *C. sporogenes* (Figure 5J). In contrast, mice receiving WT *C. sporogenes* displayed improved glucose tolerance, with lower glycemia at multiple time points and a significantly reduced GTT AUC compared with mice receiving the *fldC*-deficient strain (Figure 5K). During the pyruvate tolerance test, WT-treated mice also showed lower glycemia at intermediate time points; however, the reduction in overall PTT AUC did not reach statistical significance (Figure 5L). Thus, the glucose-tolerance phenotype associated with *fldC*-dependent *C. sporogenes* metabolism extends to WD-fed mice.

**Figure 5.**
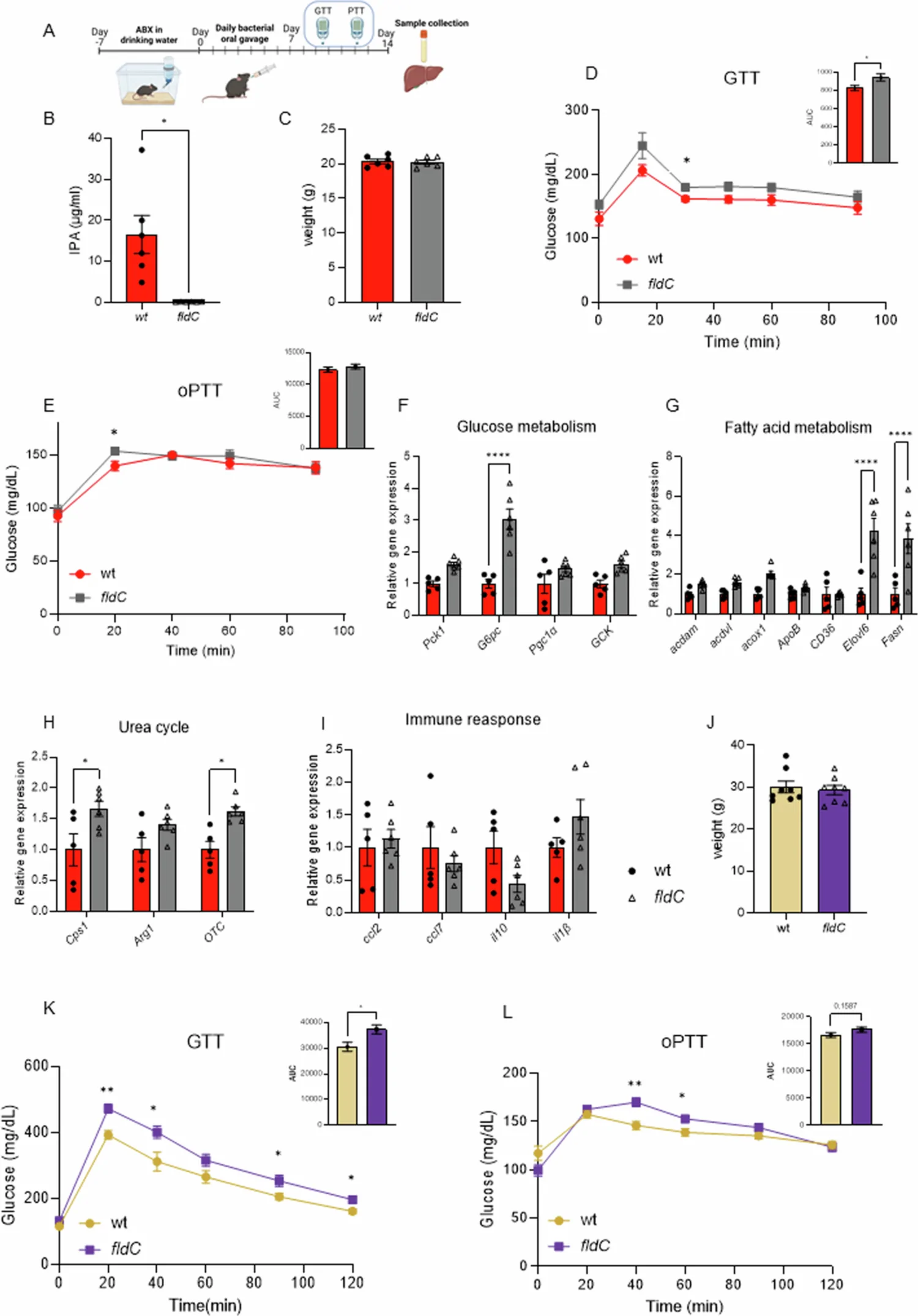
Comparison of glucose homeostasis in mice receiving wild-type or *fldC*-deficient *C. sporogenes*. (A) Experimental scheme. Mice received broad-spectrum antibiotics in drinking water for 7 days, followed by daily oral gavage with approximately 8 × 10^9^ wild-type (WT) or fldC-deficient *C. sporogenes* bacteria. Gavage continued for at least 1 week before metabolic testing and until sacrifice. Panels B–I show chow-fed mice; panels J–L show Western diet (WD)-fed mice. The WD experimental timeline is shown in Figure S4B. (B) Serum IPA concentrations following administration of WT or fldC-deficient *C. sporogenes* (*n* = 6 per group). (C) Body weight of chow-fed mice (*n* = 6 per group). (D) Intraperitoneal glucose tolerance test (GTT; 2 g/kg glucose), with area under the curve (AUC) shown in the inset (*n* = 5 per group). (E) Oral pyruvate tolerance test (oPTT; 2.5 g/kg pyruvate), with AUC shown in the inset (*n* = 7 per group). GTT and oPTT in chow-fed mice were performed in separate cohorts. (F–I) Relative hepatic mRNA expression of genes involved in glucose metabolism (F), fatty acid metabolism (G), the urea cycle (H), and immune responses (I), measured by RT-qPCR in samples collected under ad libitum feeding conditions. (J) Body weight of WD-fed mice (*n* = 8 per group). (K–L) GTT (K) and oPTT (L) in WD-fed mice (*n* = 8 per group), with AUC shown in the inset. All tolerance tests were performed following overnight fasting, during which mice received 0.25% (w/v) tryptophan in drinking water. WT and fldC-deficient groups are shown in red and gray, respectively, for chow-fed mice, and yellow and purple, respectively, for WD-fed mice. Data are presented as mean ± SEM. Individual points represent individual mice. Statistical analyzes were performed using unpaired t-tests with Welch’s correction (B, C, J), multiple unpaired t-tests comparing treatment groups at individual time points (D–E, K–L), or two-way ANOVA followed by Sidak’s multiple-comparisons test (F–I). *, *P* < 0.05; **, *P* < 0.01; ***, *P* < 0.001; ****, *P* < 0.0001.

We next examined hepatic gene expression in ad libitum-fed mice collected at the end of the recolonization experiments. This sampling condition was selected because endogenous microbial IPA production depends on dietary substrate availability and may therefore be more apparent in the fed state. In chow-fed mice, treatment with wild-type *C. sporogenes* altered the expression of selected genes involved in glucose metabolism (Figure 5F), fatty acid metabolism (Figure 5G), urea cycle function (Figure 5H), and immune responses (Figure 5I), but the overall pattern did not indicate a uniform suppression of the hepatic gluconeogenic program. We similarly examined these pathways in WD-fed mice receiving wild-type or *fldC*-deficient *C. sporogenes*. Despite the clearer improvement in glucose tolerance observed under WD conditions, hepatic expression of genes involved in glucose metabolism, fatty acid metabolism, the urea cycle, and inflammatory responses showed only limited differences between the two groups, without evidence of broad coordinated transcriptional remodeling (Fig. S4C-F). These transcriptional profiles also differ from those observed following direct IPA administration in WD-fed mice, in which animals were fasted overnight, injected with IPA the following day, and sacrificed two hours after the final injection. The differences across these in vivo paradigms may reflect the distinct nutritional states and modes of IPA exposure: continuous microbial production during ad libitum feeding versus acute pharmacological administration after fasting, as well as systemic regulatory responses that are absent from isolated hepatocytes. Importantly, the lack of a consistent transcriptional signature across these models further suggests that changes in canonical hepatic gene expression are unlikely to fully account for the effects on glucose homeostasis.

These experiments associate fldC-dependent C. sporogenes metabolism with higher circulating IPA and improved glucose tolerance. However, fldC disruption affects broader aromatic amino-acid metabolism, and equivalent intestinal abundance of the two strains was not quantitatively established. The bacterial comparison therefore cannot attribute the phenotype specifically to IPA. The complementary experiments with purified IPA demonstrate its capacity to influence glucose metabolism under the tested conditions, but do not establish its contribution relative to other factors in the recolonization model.

## Materials and methods

### Reagent

IPA (sigma-Aldrich, 220027), Indole (sigma-Aldrich, I3408), ICA (sigma-Aldrich, 284734), IAA (sigma-Aldrich, I5148), tryptamine (chemcruz, sc-206065), IBA (sigma-Aldrich, I5386), Dexamethasone (Sigma, D4902), Glucagon (Sigma, G2044), insulin (Sigma, I-2643), sodium pyruvate solution 100 mM (Biological Industries, 03-041-1B). Sodium l-lactate (Sigma, L7022-10G). l-glutamine solution (Biological Industries, 03-020-1B). DMEM high glucose (Biological Industries, 01-052-1 A). DMEM low glucose (Biological Industries, 01-050-1 A). DMEM with no glucose, no glutamine, and no sodium pyruvate (Biological Industries, 01-057-1 A). Glucose (GO) assay kit (Sigma, GAGO20-1KT). Liberase Research Grade (Sigma, 5401119001). Lipofectamine RNAiMAX Transfection Reagent (Thermo Scientific, 13778075). Fetal bovine serum (Biological Industries, 04-007-1A). Lipofectamine RNAiMAX Transfection Reagent (Thermo Scientific, 13778075).

### Animals

6–8 weeks old C57BL/6JOlaHsd males were purchased from Envigo, and housed under specific pathogen-free (SPF) conditions. Mice were allowed to acclimate to the anima facility for at least 3 days before any procedure. The mice were kept in environments with 40–70% relative humidity, ambient temperature of 22(± 2) °C and a 12 h/12 h light/dark period. Cages were enriched with a plastic tube, wooden sticks, and cotton. Where indicated, mice were fed high fat/high sucrose Western diet (Teklad, TD.08811). All animal procedures were approved by the Hebrew University of Jerusalem IACUC (animal protocols: MD-22-16959, MD-23-17187 and HU-26-18185). IPA was dissolved in DMSO to a concentration of 100mg/ml. The working solution was prepared by mixing 10% IPA stock or DMSO, 10% Tween 80, and 80% PBS. The solution was administered at 50 mg/kg body weight. Mice were assigned to experimental groups by body-weight matching to obtain comparable mean baseline body weights across groups; allocation was not randomized. Investigators were aware of group allocation during the conduct of the experiments, outcome assessment, and data analysis. No a priori formal sample size calculation was performed. Animals were excluded if they exhibited signs of poor health or injury or failed to gain weight during the pre-intervention period. Mice were deeply anesthetized with isoflurane Primal Critical Care, USA) using an open-drop method. Liquid isoflurane was applied to an absorbent paper towel placed inside a sealed desiccator, with a physical barrier preventing direct contact between the animals and the isoflurane-soaked material. Adequate anesthetic depth was confirmed by the absence of a pedal withdrawal response to toe pinch. For terminal tissue collection, blood was collected by cardiac puncture while mice remained deeply anesthetized, followed immediately by tissue harvesting, with death ensured by exsanguination and removal of vital organs. For primary hepatocyte isolation, the inferior vena cava was cannulated and the portal vein was transected under deep anesthesia to permit liver perfusion, resulting in exsanguination; animals did not recover from anesthesia.

### Glucose and pyruvate tolerance tests

Mice were fasted overnight. For bacterial recolonization experiments, mice received 0.25% (w/v) tryptophan in their drinking water during the overnight fasting period. For PTT, mice were orally gavaged with a 25% pyruvate solution in Dulbecco PBS (dPBS) at a dose of 2.5 g/kg. For GTT, mice were injected i.p. with a 20% D-glucose solution in dPBS at a dose of 2 g/kg. Blood glucose levels were measured at the indicated time points following injection.

### Antibiotic administration

1g/L ampicillin(Formedium), 1g/L Metronidazole(Sigma-aldrich, M3761), 1g/L Neomycin sulfate(goldbio 1405-10-3), 0.5g/L vancomycin (ApexBio B1223) were administered in the drinking water for a week.

### *C. sporogenes* colonization

*Clostridium sporogenes* wild-type (WT) and *fldC* mutant strains were kindly provided by the Sonnenburg lab.^33^ Bacteria were cultured in an anaerobic chamber (COY labs, USA) overnight in TYG medium (30 g/L tryptone, 20 g/L yeast extract, 1 g/L sodium thioglycolate). Cultures were supplemented with glycerol to a final concentration of 30% and stored at −80 °C. Following antibiotic treatment, mice were orally gavaged with approximately 8 × 10^9^ bacteria daily for at least one week before metabolic testing and until sacrifice. To assess bacterial abundance, fresh stool samples from each cage were inoculated into TYG medium and incubated overnight under anaerobic conditions. The following day, bacterial cultures were centrifuged at 10,000× g for 10 minutes, and the resulting pellets were resuspended in 500 μL DDW. Samples were then heated at 95 °C for 10 minutes to lyse the bacteria and release DNA. PCR amplification was performed using specific primers as described^33^and the Phanta DNA polymerase enzyme, according to the manufacturer’s instructions. PCR products were analyzed by electrophoresis on a 1% agarose gel.

### Primary mouse hepatocyte isolation and culture conditions

Primary hepatocytes were isolated using a Liberase perfusion technique. Eight-week-old WT mice were sacrificed by Isoflurane inhalation, and the liver was perfused via the vena cava with HBSS (0.5 mM EDTA, 25 mM HEPES, calcium-free, magnesium-free, phenol red-free; Sartorius Israel, 02-018-1A). During perfusion, the portal vein was clamped for 5 seconds after being cut. Subsequently, 12.5 mL of digestion buffer containing 25μg/ml Liberase was perfused. The liver was excised, rinsed with 10 mL of dPBS (Sartorius Israel, 02-023-1A), and gently scraped. The tissue was suspended in 20 mL of plating medium and passed through a 70- μm cell strainer. Live hepatocytes were separated using a Percoll gradient and plated in plating medium (high-glucose DMEM supplemented with 10% FBS, 2 mM pyruvate, 1% penicillin/streptomycin, 0.1 μM insulin, and 1 μM dexamethasone). After 3-4 hours the medium was replaced with maintenance medium (low-glucose DMEM supplemented with 0.2% BSA, 4 mM L-glutamine, 1 mM pyruvate, 1% penicillin/streptomycin, 1 nM insulin, and 0.1 μM dexamethasone). For siRNA-mediated suppression of PXR, cells were transfected with siRNA oligos targeting *Nr1i2* (purchased for IDT) or a non-targeting control according to the manufacturer’s instructions. After 48 hours of incubation, knockdown efficiency was assessed by quantitative PCR.

### Hepatic glucose production

Day after isolation, primary hepatocytes were incubated for 3 hours with hormonal starvation medium (low-glucose DMEM supplemented with 0.2% BSA, 4 mM L-glutamine, 1 mM pyruvate, 1% penicillin/streptomycin) containing treatment. Following that the cells washed with dPBS and the media was replaced to DMEM devoid of glucose and phenol red (Sigma D-5030), supplemented with 0.2% BSA, sodium bicarbonate, and 1% Penicillin/Streptomycin. Additionally, the medium contained either sodium pyruvate and sodium L-lactate(2 mM and 20 mM, respectively) Glycerol (10 mM) or Glutamine (4 mM) as gluconeogenic substrates. Cells were exposed to these conditions for 6 hours with glucagon (100 nM) stimulation. For the glucose quantification, 50 μl of the cell culture media was collected and mixed with 100 μl of the Glucose Oxidase (GO) Assay Kit (Sigma, GAGO20-1KT). The samples were then incubated at 37 °C for 30 minutes. To terminate the reaction, 100 μl of 12 N sulfuric acid was added. The absorbance of the resulting glucose was measured at 540 nm. Glucose levels were normalized to total protein.

### Gene expression

In the morning, culture medium was replaced with hormonal starvation medium containing the indicated treatment for 3 h. Cells were then stimulated with glucagon (100 nM) for an additional 3 h before harvesting.

### RNA extraction and cDNA preparation

Total RNA was isolated using one of two methods. Cell culture total RNA was isolated using Nucleospin RNA, mini kit for RNA purification (Macherey-Nagel, #740955.50), according to the manufacturer’s protocol. Total RNA from liver tissue was extracted using TRIzol (or TRI reagent). Subsequently, cDNA was synthesized using a qScript cDNA Synthesis Kit (Quantabio, #95047-500) following the manufacturer’s instructions.

### Quantitative real-time polymerase chain reaction (qPCR)

qPCR was performed with a C1000 Touch thermal cycler CFX384 instrument (Bio-Rad) using Luna Universal qPCR master mix (NEB, M3003E). All qPCR experiments were replicated at least three independent times. Gene values were normalized with *b2M* and *Rpl13*. Bio-Rad CFX Maestro software was used to analyze gene expression.

### Metabolite extraction for polar metabolite analysis

Extraction and analysis of polar metabolites and lipids were conducted following previously established methods,^34^ ^,^ ^35^ with minor modifications. Mouse primary hepatocytes (2 × 10^6^ cells), treated with indole-3-propionic acid (IPA) for 3 hrs or with DMSO (control), were extracted with 1 mL of pre-chilled (−20 °C) MTBE:methanol (1:3, v/v). Samples were vortexed briefly, sonicated for 30 min in an ice-cold bath (with intermittent vortexing every 10 min), and then mixed with 0.5 mL of DDW:methanol (3:1, v/v) containing labeled amino acid standards (^13^C and ^15^N; Sigma, 767964; 1:500 dilution). After thorough vortexing and centrifugation, the upper organic phase was collected. The remaining polar phase was extracted using an additional 0.5 mL MTBE. Polar phase extracts were collected and dried under a gentle nitrogen stream, and stored at −80 °C.

### LC-MS analysis of polar metabolites

Dried polar extracts were reconstituted in 120 μL methanol:DDW (50:50, v/v), centrifuged twice, and 70 μL was transferred to UPLC vials. Analysis utilized an Acquity I-class UPLC system (Waters) coupled with a Q Exactive Plus Orbitrap mass spectrometer (Thermo Fisher Scientific) operating in negative ionization mode. Metabolites were separated using a SeQuant Zic-pHilic column (150 × 2.1 mm) with a corresponding guard column (Merck). Mobile phases comprised ACN (phase B) and 20 mM ammonium carbonate with 0.1% ammonium hydroxide in DDW:ACN (80:20, v/v; phase A). The flow rate was 200 μL/min at 45 °C. The gradient was: 75% B (0–2 min), decreasing to 25% B by 14 min, holding until 18 min, returning to 75% B at 19 min, and equilibration until 23 min. Injection volume was 2 μL. Mass spectra were acquired in negative heated electrospray ionization (HESI) mode over the mass range 70–1050 m/z. Source parameters: capillary temperature 325 °C, spray voltage 3.25 kV, sheath gas 40 units, auxiliary gas 10 units, auxiliary gas temperature 50 °C. MS1 spectra were acquired at a 35,000 FWHM resolution, and MS/MS data collection was performed at a 17,500 FWHM resolution (3 m/z isolation window). Polar data processing was done using Progenesis QI (Waters). identifying compounds based on accurate mass (≤5 ppm), retention time, and spectral libraries generated from injected standards. Relative polar metabolite levels were normalized to the internal standards, and cell number.

### ^13^C-lactate tracing

Primary mouse hepatocytes were treated with IPA or vehicle for 3 h in hormonal starvation medium. Cells were then incubated for 3 h, in the absence or presence of glucagon, in glucose-production medium containing uniformly labeled [U-^13^C]lactate (Cambridge Isotope Laboratories) and the corresponding IPA or vehicle treatment. Following incubation, cells were washed with ice-cold PBS, and intracellular metabolites were extracted under cold conditions using 80% methanol. Polar metabolites were analyzed by ion chromatography-high-resolution mass spectrometry (IC-HRMS) using a Dionex Integrion HPIC system coupled to a Q Exactive Plus hybrid Fourier-transform mass spectrometer equipped with a heated electrospray ionization source (Thermo Fisher Scientific). The IC system comprised an isocratic pump, an online KOH eluent generator, a thermostatted compartment, an autosampler, an acetonitrile make-up pump, an electrolytic suppressor operated in external-water mode, and a conductivity detector. Anionic metabolites were separated on a hydroxide-selective anion-exchange column (Dionex IonPac AS11-HC, 250 × 2.1 mm; Thermo Fisher Scientific). The mass spectrometer was operated in negative electrospray-ionization mode with a spray voltage of 3 kV, capillary temperature of 300 °C, sheath-gas setting of 40 arbitrary units, and auxiliary-gas setting of 10 arbitrary units. Full-scan spectra were acquired over an m/z range of 50-600 at a resolving power of 140,000. The IC–MS system was controlled and data were processed using Xcalibur software. Incorporation of lactate-derived carbon was quantified from the isotopologue distributions of glucose-6-phosphate and tricarboxylic acid-cycle intermediates after correction for natural isotope abundance.

### Targeted measurement of IPA in serum and liver

IPA concentrations in serum and liver were determined by targeted liquid chromatography-tandem mass spectrometry (LC-MS/MS) using a Nexera binary ultrahigh-performance liquid chromatography system (Shimadzu) coupled to a QTRAP 6500+ triple-quadrupole mass spectrometer (SCIEX). Serum samples (5-10 μl) were extracted with 80% methanol containing 10 ng/mL d₄-indoleacetic acid as an internal standard. Liver samples were homogenized with 3-mm zirconium oxide beads in 1 mL of methanol/water (1:1, v/v) containing 0.1% formic acid and the internal standard. Extracts were centrifuged at 10,000 rpm for 10 min, and the resulting supernatants were passed through 0.2-μm regenerated-cellulose membrane filters (Teknokroma, Spain) before analysis. Chromatographic separation was performed on an Acclaim C18 column (2.1 × 150 mm, 2.2 μm; Thermo Fisher Scientific) using a linear acetonitrile/water gradient containing 0.1% acetic acid. IPA was detected by electrospray ionization in multiple-reaction-monitoring mode. Calibration standards were prepared from authentic IPA (Sigma-Aldrich) at concentrations of 0.1, 0.25, 1, 2, 5, 10, 25, and 100 ng/mL, each containing 10 ng/mL internal standard. IPA concentrations were calculated from the calibration curve using the analyte-to-internal-standard peak-area ratio and normalized to serum volume or liver tissue weight, as appropriate.

### Western blot

Cells were incubated in hormonal starvation medium containing the indicated treatments for 3 hours. Following this, the medium was replaced with fresh hormonal starvation medium containing the same treatments and either glucagon (100 nM) or insulin (20 nM) for 30 minutes. After treatment, cells were harvested in RIPA buffer supplemented with protease inhibitors and shaken at 4 °C for 15 minutes. Cells were then scraped and transferred to Eppendorf tubes, followed by centrifugation at maximum speed at 4 °C for 15 minutes. The supernatant was collected into new Eppendorf tubes. Protein concentration was determined, and 18 μg of protein from each sample was separated by 10% SDS-PAGE and transferred to PVDF membranes (Millipore IPVH00010). Membranes were blocked and incubated overnight at 4 °C with the following primary antibodies: CREB (CST, 9104S), *p*-CREB (CST, 9198S), AKT (CST, 2920S), *p*-AKT (CST, 4060S), AMPK (CST, 5831), *p*-AMPK (CST, 2535), and Vinculin (CST, 13901). After washing with TBST, membranes were incubated with peroxidase-conjugated secondary antibodies in 5% skim milk at room temperature for 1 hour. Protein signals were detected using enhanced chemiluminescence substrate (ECL, Bio-Rad).

### Intracellular NAD^+^/NADH

Intracellular NAD⁺ and NADH levels were measured using an enzymatic cycling assay as previously described.^36^ Briefly, 2.5 × 10^6^ cells were seeded in 10-cm culture dishes. Cells were treated with IPA for 3 hours, followed by stimulation with glucagon for an additional 3 hours. During the final 1 hour of incubation, either rotenone (25 nM) or oligomycin (2 μM) was added to the culture medium. Following treatment, cells were washed with cold dPBS, scraped with 5 ml dPBS, and centrifuged at 1,000× g for 5 minutes at 4 °C. The cell pellet was resuspended in 500 μl extraction buffer (20 mM nicotinamide, 20 mM NaHCO₃, 100 mM Na₂CO₃) and centrifuged at 1,200× g for 15 minutes at 4 °C. For NADH determination, 250 μl of the supernatant was incubated at 60 °C for 30 minutes to decompose NAD⁺. Both heated (NADH) and unheated (total NAD) samples were used for analysis. A reaction mixture containing 160 μl NAD-cycling buffer (100 mM Tris-HCl, pH 8.0; 0.5 mM thiazolyl blue; 1 mM phenazine ethosulfate; 1% BSA) and 5 unit alcohol dehydrogenase (ADH) was added to each well of a 96-well plate containing 20 μl of sample. After incubation for 1 minute at 30 °C in the dark, absorbance at 570 nm was measured. Subsequently, 20 μl of 6 M ethanol was added to initiate the cycling reaction, and the change in absorbance at 570 nm was recorded every 30 seconds for 10 minutes at 30 °C using a plate reader. All samples were analyzed in triplicate. NAD⁺ levels were calculated by subtracting NADH values from total NAD measurements.

### Metabolic flux analysis (Seahorse)

Mouse primary hepatocytes were seeded at a density of 7,500 cells/well in XF96 cell culture plates (Agilent Technologies) in plating medium and allowed to attach for 3 h at 37 °C and 5% CO₂. The plating medium was then replaced with maintenance medium, and cells were incubated overnight. The following morning, cells were treated with IPA (50-500 μM) for 3 h in hormonal starvation medium (maintenance medium without insulin and dex). Cells were subsequently treated with 100 nM glucagon for an additional 2 h in the continued presence of IPA. The Seahorse assay medium consisted of DMEM supplemented with 5 mM HEPES, 0.2% BSA, 1% penicillin/streptomycin, 2 mM sodium pyruvate, and 20 mM sodium lactate, and was adjusted to pH 7.4. The final volume was 180 μL/well. During the final hour of glucagon treatment, cells were incubated at 37 °C in a non-CO₂ incubator for pre-equilibration. Oxygen consumption rate (OCR) was measured using an XFe96 Extracellular Flux Analyzer (Agilent Technologies). A mitochondrial stress test was performed by sequential injection of 1 μM oligomycin, 1 μM FCCP, and 0.5 μM antimycin A together with 0.5 μM rotenone. Following the assay, cell number was determined using the CyQUANT Cell Proliferation Assay Kit (Thermo Fisher Scientific), and OCR values were normalized to cell number.

### MTT

Mouse primary hepatocytes were seeded at 7,500 cells/well in 96-well plates and allowed to attach for 3 h at 37 °C and 5% CO₂. The plating medium was then replaced with hormonal starvation medium containing increasing concentrations of IPA (10–500 μM) or sodium azide (2.5 mM) as a positive control, and cells were treated overnight. Subsequently, 20 μL of MTT solution (5 mg/mL) was added to each well and incubated for 1 h at 37 °C. The medium was then aspirated, 200 μL of isopropanol was added to each well, and the plate was shaken for 5 min. Absorbance was measured at 560 nm, with background absorbance measured at 670 nm and subtracted from the 560 nm readings.

### Mitochondrial isolation and CPS1 activity

A total of 2.5 × 10^6^ cells were seeded in 10-cm culture dishes. Cells were treated with IPA for 3 hours, followed by stimulation with glucagon for an additional 3 hours. Following treatment, cells were washed with ice-cold dPBS, scraped with 5 mL dPBS, and centrifuged at 1,000× g for 5 minutes at 4 °C. The resulting cell pellet was resuspended in 1 mL ice-cold hypotonic Tris buffer (10 mM, pH 7.6) and transferred to 2 mL microcentrifuge tubes. After a 3-minute incubation on ice, cells were homogenized using a homogenizer (1,000 rpm, 15 strokes). Subsequently, 200 μL of 1.5 M sucrose was added to the homogenate, followed by centrifugation at 600× g for 10 minutes at 4 °C. The supernatant was collected and transferred to a new tube, then centrifuged at 14,000× g for 10 minutes at 4 °C to pellet mitochondria. The supernatant was discarded, and the mitochondrial pellet was resuspended in 200 μL of 10 mM Tris buffer (pH 7.6).

For CPS1 activity measurement,^37^ 5 μg of mitochondrial protein was incubated in a final volume of 100 μL containing assay buffer (50 mM HEPES, pH 7.0; 12.5 mM KHCO₃; 0.75 mM (NH₄)₂SO₄; 5 mM MgCl₂; 50 mM KCl; 1 mM TCEP; 0.01% Triton X-100; 0.0025% BSA; 0.2 mM *N*-acetylglutamate; 5 mM ATP). Samples were incubated at 37 °C for 1 hour. Following incubation, 40 μL of the reaction mixture was transferred to a PCR tube, and 2 μL of 2 M hydroxylamine was added. Samples were heated at 95 °C for 10 minutes. Equal volumes of solution A (0.85 g antipyrine dissolved in 40% H₂SO₄) and solution B (0.625 g diacetyl monoxime dissolved in 5% acetic acid) were mixed, and 160 μL of this mixture was added to each sample. The samples were then incubated at 95 °C for 15 minutes. After cooling to room temperature, absorbance was measured at 458 nm using a plate reader. Mitochondrial protein samples were analyzed by Western blotting to assess CPS1 protein abundance following treatment, using antibodies against CPS1 (proteintech 18703-1-AP) and SDHA (CST #11998S).

### Urea detection

A day after seeding, cells were treated with IPA or DMSO for 3 hours under hormonal starvation conditions. Following starvation, cells were washed twice with DPBS and incubated for 6 hours in phenol red–free DMEM-based assay medium supplemented with 8.3 mg/mL DMEM powder, 3.7 mg/mL sodium bicarbonate, 0.2% BSA, 5 mM glucose, 1% penicillin–streptomycin, 2 mM sodium pyruvate, 2 mM L-glutamine, and 1 mM NH₄Cl, in the presence or absence of IPA and glucagon. Urea concentration was measured in cell culture supernatants, which were collected after the incubation period, and in serum samples, using a commercially available assay kit (abcam, ab234052) according to the manufacturer’s instructions. Urea levels were normalized to total protein.

### Triglyceride detection

Approximately 50 mg of liver tissue was homogenized in 5% NP-40 using bead beating. Samples were then heated to 100 °C and cooled to room temperature twice, followed by centrifugation at maximum speed for 2 minutes. The resulting supernatants were collected and diluted 1:10 prior to analysis. Serum samples were diluted 1:5. Triglyceride concentration was measured using a commercially available assay kit (abcam, ab65336) according to the manufacturer’s instructions.

### ATP measurement

Cells were seeded in 24-well plates and, the following day, the medium was replaced with hormonal starvation medium containing IPA or DMSO for 3 hours. After starvation, the medium was replaced with fresh medium containing the indicated treatments (IPA and glucagon). ATP levels were measured using a commercially available assay kit (Promega, G9241) according to the manufacturer’s instructions.

### Statistical analysis

Data are presented as mean ± SEM. Statistical comparisons were performed using GraphPad Prism 9.0. ANOVA with Sidak post hoc analysis or an unpaired two-tailed Student's t-test was used. A *p*-value of < 0.05 was considered statistically significant.

## Discussion

In this study, we identify indole-3-propionic acid (IPA) as a microbiota-derived indole metabolite that directly modulates hepatic glucose metabolism. IPA suppressed glucagon-stimulated glucose production in primary hepatocytes in a substrate-selective manner, with a stronger effect on pyruvate/lactate- and glutamine-supported glucose production than on glycerol-supported glucose production. This effect occurred without detectable inhibition of proximal glucagon-CREB or insulin-AKT signaling and was not prevented by perturbation of the canonical IPA-associated receptors AhR or PXR/*Nr1i2*. Mechanistically, IPA reduced the incorporation of lactate-derived carbon into gluconeogenic and mitochondrial intermediates, altered cellular NAD⁺/NADH in a manner requiring intact, rotenone-sensitive Complex I-dependent NADH oxidation, increased ATP availability, and attenuated CPS1-linked urea cycle activity during glucagon stimulation. In vivo, short-term IPA administration improved glucose handling in WD-fed mice, while *fldC*-dependent microbial IPA production modestly improved glucose tolerance following microbiome depletion and recolonization. Together, these findings support a model in which IPA acts as a gut microbiota-derived metabolic effector that tunes hepatocyte mitochondrial metabolic state and thereby limits hepatic glucose output.

A central feature of the IPA response was its substrate selectivity. IPA robustly suppressed glucose production from pyruvate/lactate, while glycerol-supported glucose production was largely preserved. Because glycerol enters the gluconeogenic pathway downstream of mitochondrial pyruvate metabolism, this pattern argues against a nonspecific inhibition of gluconeogenesis or a general impairment of hepatocyte glucose-producing capacity. The additional suppression of glutamine-supported glucose production further supports the idea that IPA preferentially affects substrates that require mitochondrial metabolism, anaplerotic processing, or mitochondrial-cytosolic exchange before contributing to glucose synthesis. Although IPA also attenuated glucagon-induced *Pck1* and *G6pc* expression in primary hepatocytes, this transcriptional effect alone does not fully explain the functional phenotype. Glycerol-supported glucose production remained largely intact despite reduced expression of these genes, and the in vivo transcriptional profile did not show coordinated suppression of the gluconeogenic program. Thus, while IPA can influence selected glucagon-responsive transcripts, the dominant functional effect appears to involve metabolic control of substrate handling rather than broad transcriptional repression of gluconeogenesis.

The concentrations used in the mechanistic experiments should be distinguished from typical endogenous human exposure. IPA at 50 μM exceeds typical fasting human circulating concentrations of approximately 1 μM by about 50-fold.^19^ ^,^ ^31^ ^,^ ^32^ ^,^ ^38^ We selected 50 μM to obtain a robust and reproducible response for mechanistic characterization. The expanded dose-response experiment detected significant suppression of glucose production at 25 μM, with greater effects at higher concentrations (Fig. S1B). Although endogenous serum IPA in refed chow-fed mice reached approximately 32-37 μM in one cohort (Figure 4B), these measurements provide species- and nutritional-state-specific context and do not establish that 50 μM represents typical physiological exposure in humans. The present findings therefore demonstrate a direct effect of IPA on hepatocyte glucose production under the tested conditions, while its contribution to hepatic glucose regulation at endogenous human concentrations remains to be established.

The isotope tracing and metabolomics data provide further insight into this metabolic control. IPA reduced the fraction of ^13^C₃-labeled G6P from [U-^13^C]-lactate, particularly under glucagon-stimulated conditions, directly linking the reduction in glucose output to diminished incorporation of lactate-derived carbon into the gluconeogenic pathway. IPA also reduced ^13^C₂ enrichment in multiple TCA cycle intermediates, including citrate, *α*-ketoglutarate, fumarate, and malate. The coordinated nature of this reduction argues against inhibition at a single TCA cycle step and instead suggests altered entry, retention, or propagation of lactate-derived carbon within mitochondrial metabolism. Notably, these tracing results occurred alongside increased steady-state abundance of selected TCA- and urea cycle-associated metabolites, including citrulline, argininosuccinate, fumarate, and malate. This combination, reduced lactate-derived labeling but accumulation of selected metabolite pools, suggests that IPA does not simply suppress mitochondrial metabolism globally. Rather, IPA appears to remodel the source, turnover, or partitioning of metabolite pools at the interface between the TCA cycle, gluconeogenesis, and nitrogen metabolism.

The redox data suggest one mechanism by which IPA may alter lactate-dependent glucose production. IPA increased the cellular NAD⁺/NADH redox balance under basal conditions, and this effect was abolished by rotenone, indicating dependence on Complex I-linked mitochondrial electron transport. Glucagon also increased NAD⁺/NADH redox balance, consistent with activation of oxidative metabolism during gluconeogenic stimulation. However, IPA did not simply enhance this glucagon response. Instead, the combination of IPA and glucagon shifted the NAD⁺/NADH ratio toward baseline, indicating that IPA changes the characteristic redox response to glucagon. Since lactate-supported gluconeogenesis requires coordinated cytosolic and mitochondrial redox handling, altered NADH oxidation or redox coupling could limit efficient conversion of lactate-derived carbon into glucose even when upstream glucagon signaling remains intact. Our rotenone experiments indicate that the IPA-sensitive redox response requires intact rotenone-sensitive electron transport. However, neither the inhibitor data nor the OCR measurements identify Complex I as a direct molecular target of IPA. Rather, these findings support a model in which IPA modifies the mitochondrial metabolic state that accompanies glucagon stimulation. The increase in ATP levels, particularly during glucagon stimulation, further argues that IPA does not globally inhibit mitochondrial energy metabolism. IPA appears to induce a distinct mitochondrial metabolic state in which respiration-linked redox handling and energy availability are altered, but the routing of lactate-derived carbon into gluconeogenesis is reduced.

The CPS1 and urea-cycle findings extend this model beyond carbon metabolism alone. Untargeted metabolomics pointed to selective remodeling of metabolites linked to nitrogen handling, and IPA attenuated glucagon-stimulated CPS1 activity and urea secretion without a detectable reduction in total CPS1 protein. These data suggest that the reduction in CPS1-dependent metabolism is unlikely to reflect loss of enzyme abundance and may instead result from altered mitochondrial metabolic state, or post-translational regulation of CPS1 activity. The molecular basis of this activity change remains unresolved. Pharmacological inhibition of CPS1 reduced glucagon-stimulated glucose production and partially phenocopied the effect of IPA, but IPA retained activity under CPS1 inhibition, indicating that CPS1 is not the sole route through which IPA limits glucose output. We therefore interpret CPS1 activity as one functional node within the broader glucagon-stimulated mitochondrial program that supports lactate-dependent hepatic glucose production, rather than as the direct molecular target of IPA.

The IPA administration experiments demonstrate improved glucose handling following short-term pharmacological exposure, while highlighting the rapid nature and context dependence of the response. Previous studies have shown that IPA can improve metabolic,^20^ ^,^ ^22^ inflammatory,^24^ intestinal,^21^ ^,^ ^22^ neurological,^26^ or cardiovascular outcomes^23^ in vivo, but these effects have generally been examined after repeated exposure over days to weeks. For example, IPA improved fasting glucose, fasting insulin, and HOMA-IR in rats after six weeks of dietary supplementation,^20^ and other studies have used repeated IPA administration or dietary supplementation over approximately 10 days to several weeks. In contrast, our IPA treatment paradigm was deliberately short, consisting of once-daily IPA administration for only five days in WD-fed mice. The circulating concentration measured 2 h after injection substantially exceeded both endogenous IPA concentrations and those used in the hepatocyte experiments. This single measurement does not establish the peak concentration or define the exposure profile, and hepatic IPA concentrations were not measured following administration. Despite this limited exposure window, IPA reduced fasting glucose and improved glucose handling during both GTT and PTT. These effects occurred without marked changes in body weight, serum or hepatic triglycerides, or coordinated transcriptional remodeling of hepatic lipid, inflammatory, urea-cycle, or gluconeogenic gene programs. Indeed, the hepatic transcriptional response in vivo differed from that observed in primary hepatocytes, reinforcing the idea that transcriptional repression of gluconeogenesis is unlikely to be the dominant mechanism underlying the physiological phenotype. The divergent transcriptional response remains unexplained. Differences in hormonal regulation, peripheral glucose disposal, glycogen metabolism, or other compensatory responses may contribute, but these possibilities were not directly tested. Instead, the rapid improvement in fasting glycemia and reduced PTT AUC are more consistent with acute modulation of hepatic metabolic state or flux regulation. The *C. sporogenes* recolonization experiments provide complementary evidence linking *fldC*-dependent microbial metabolism to host glucose homeostasis. In chow-fed mice, administration of wild-type *C. sporogenes* was associated with higher circulating IPA levels and modestly improved glucose tolerance compared with the *fldC*-deficient strain, whereas pyruvate tolerance was largely unchanged. Notably, the glucose-tolerance phenotype was also observed in WD-fed mice, in which wild-type *C. sporogenes* improved glucose tolerance and reduced glycemia at several time points during the pyruvate tolerance test. These findings suggest that the metabolic consequences of *fldC*-dependent microbial activity may become more apparent under diet-induced metabolic stress. However, because the *fldC* pathway influences additional products of aromatic amino-acid metabolism,^33^ and equivalent intestinal abundance of the two strains was not quantitatively established, these experiments cannot attribute the phenotype exclusively to IPA. Rather, their convergence with the direct effects of purified IPA in primary hepatocytes and WD-fed mice supports IPA as a contributor to the observed metabolic phenotype. Potential changes in upstream metabolites, including indole-3-lactate, phenyllactate, and hydroxyphenyllactate,^33^ may also contribute to differences between the strains. These metabolites were not quantified in our experiments, so their contribution to the metabolic phenotype remains unresolved. Together, these findings suggest that the impact of microbiota-derived IPA on glucose homeostasis is influenced by nutritional and metabolic context.

The effects of several compounds in our screen suggest that the regulation of hepatocyte glucose production by indole derivatives extends beyond IPA. Together with our previous structure-activity studies identifying the indole moiety as necessary for SR-18292 activity,^29^ these findings support further investigation of indole-containing compounds as candidates for modulating hepatic glucose metabolism. However, the shared scaffold encompasses functionally diverse molecules, including serotonin, and does not imply a common mechanism or uniformly beneficial metabolic effects. Future studies should define how individual structural features influence substrate selectivity, molecular targets, exposure, and safety. Within this broader opportunity, IPA provides a biologically motivated starting point because of its microbial biosynthesis and repeated associations with metabolic health in human studies. Based on these findings, we propose that IPA links gut microbial metabolism to hepatic glucose regulation by altering the mitochondrial metabolic state of hepatocytes. In this model, IPA promotes respiration-dependent redox remodeling, modifies the handling of lactate-derived carbon within mitochondrial and gluconeogenic pathways, and attenuates glucagon-stimulated CPS1-linked urea-cycle activity. These coordinated effects reduce the capacity of mitochondria-dependent substrates to support hepatic glucose production without globally inhibiting gluconeogenesis, disrupting proximal hormone signaling, or broadly suppressing hepatic metabolic gene expression. This model places IPA within a growing class of microbiota-derived metabolites that act not only through classical receptor-mediated pathways, but also by directly reshaping intracellular metabolic networks. From a therapeutic perspective, these findings suggest that microbial tryptophan metabolism, IPA-producing bacteria, or optimized IPA-related compounds could potentially be used to modulate excessive hepatic glucose output in metabolic disease. The relatively rapid improvement in glucose handling after short-term IPA administration further supports the possibility that this pathway may be pharmacologically or microbiologically actionable. However, several issues remain to be addressed before such approaches can be advanced, including the durability of the response, the exposure levels required to affect hepatic metabolism, the contribution of extrahepatic tissues, and whether sustained enhancement of IPA production can improve glucose homeostasis under diet-induced metabolic stress.

In summary, our findings identify IPA as a microbiota-derived regulator of hepatocyte mitochondrial metabolic state. By selectively limiting lactate- and glutamine-supported glucose production, remodeling redox and ATP balance, and attenuating CPS1-linked mitochondrial nitrogen metabolism, IPA directly modulates the metabolic programs that support glucagon-stimulated hepatic glucose output. These results provide a mechanistic framework for understanding how microbial tryptophan metabolites can influence systemic glucose homeostasis through direct effects on liver metabolism.

## Supplementary Material

Fig S2 Fig S2.tif

Fig S1 Fig S1.tif

Fig S3 Fig S3.tif

Fig S4 Fig S1.tif

KGMI_A_2760041_SM4432 KGMI_A_2760041_SM4432.docx

## Data Availability

The data supporting the findings of this study are provided with the manuscript as Supplementary Data File 1. The dataset has also been deposited in Zenodo under the reserved DOI https://doi.org/10.5281/zenodo.22654680.
