# Supplementary material for: Microbiota-derived indole-3-propionic acid regulates glucose homeostasis via remodeling of hepatic mitochondrial metabolism
Source: Gut Microbes. 2026 Nov 6;18(1):2760041. doi: 10.1080/19490976.2026.2760041 (PMC13757629; doi:10.1080/19490976.2026.2760041)
Supplement: Supplementary file 1 [file KGMI_A_2760041_SM4432.docx]

**Figure S1. Indole-derived metabolites suppress gluconeogenic genes but do not modulate hepatic signaling. (A)** Relative expression of *G6pc*, *Pck1*, and *Ppargc1a* following treatment with the indicated indole derivatives (50 μM) for 3 h, followed by an additional 3 h with or without glucagon (100 nM). Gene expression was measured by RT-qPCR. Statistical comparisons were made against the DMSO + glucagon group. **(B)** Glucose production following pretreatment with increasing concentrations of IPA (1-100 μM) for 3 h, followed by 6 h with or without glucagon (100 nM) and IPA, using pyruvate (2 mM) and lactate (20 mM) as substrates. **(C)** MTT signal following overnight treatment with IPA (10-500 μM) or sodium azide (2.5 mM), included as a positive control for reduced MTT signal. **(D–E)** Immunoblots and quantification following pretreatment with IPA (50 μM) or DMSO for 3 h and subsequent stimulation for 30 min with insulin (10 nM; D) or glucagon (100 nM; E), with IPA maintained during stimulation. Phosphorylated AKT (Ser473), CREB (Ser133), and AMPK (Thr172) signals were normalized to their respective total protein signals. Data are presented as mean ± SEM. Statistical analyses were performed using one-way ANOVA followed by Dunnett’s post hoc test for multiple comparisons. *, P < 0.05; **, P < 0.01; ***, P < 0.001; ****, P < 0.0001.

**Fig S2. Effects of AhR inhibition and PXR knockdown on hepatocyte responses to IPA. (A–B)** Primary mouse hepatocytes were treated with IPA (50 μM) or DMSO in the presence or absence of the aryl hydrocarbon receptor (AhR) inhibitor CH223191 (1 μM). Glucose production (A) and relative expression of *Pck1*, *G6pc*, and the AhR target gene *Cyp1a1* (B) were measured under basal or glucagon-stimulated conditions. **(C–D)** Primary mouse hepatocytes were transfected with control siRNA (siCtrl) or siRNA targeting Nr1i2, which encodes the pregnane X receptor (PXR), for 48 h before treatment with IPA (50 μM) or DMSO. Glucose production (C) and relative expression of *Pck1*, *G6pc*, and *Nr1i2* (D) were measured under basal or glucagon-stimulated conditions. For glucose production assays (A, C), cells were pretreated with IPA for 3 h, followed by 6 h with or without glucagon (100 nM), using pyruvate (2 mM) and lactate (20 mM) as substrates. For gene expression assays (B, D), cells were pretreated with IPA for 3 h, followed by an additional 3 h with or without glucagon (100 nM), before RT-qPCR analysis. Data are presented as mean ± SEM. Statistical analyses were performed using two-way ANOVA followed by Sidak’s post hoc test for multiple comparisons. *, P < 0.05; **, P < 0.01; ***, P < 0.001; ****, P < 0.0001; ns, not significant.

**Figure S3. (A)** Serum IPA concentration following IPA administration (i.p.) in WD-fed mice. Serum was collected 2 hrs after IPA administration in mice fasted overnight.

**Figure S4. Microbial Recolonization and Metabolic Analysis.** **(A)** PCR analysis of bacterial DNA obtained from anaerobic cultures of stool samples collected from individual cages following administration of wild-type (WT) or *fldC*-deficient *C. sporogenes*. WT and *fldC*-deficient bacterial samples were included as controls. Expected PCR product sizes were approximately 600 bp for WT and 2,800 bp for the *fldC*-deficient strain. **(B)** Experimental timeline for the Western diet (WD) recolonization study. WD feeding began 35 days before the first bacterial gavage. Mice received antibiotics during the final 7 days before gavage, followed by daily oral administration of approximately 8 × 10⁹ WT or *fldC*-deficient *C. sporogenes* bacteria. Bacterial administration continued throughout metabolic testing and until sacrifice. **(C–F)** Relative hepatic mRNA expression of genes involved in glucose metabolism (C), fatty acid metabolism (D), the urea cycle (E), and immune responses (F), measured by RT-qPCR in samples collected under ad libitum feeding conditions at the end of the experiment. WT and *fldC*-deficient groups are shown in yellow and purple, respectively. Data are presented as mean ± SEM. Individual points represent individual mice. Statistical analyses were performed using two-way ANOVA followed by Sidak’s post hoc test for multiple comparisons.
